# MDM4 Isoform Expression in Melanoma Supports an Oncogenic Role for MDM4-A

**DOI:** 10.1155/2021/3087579

**Published:** 2021-10-16

**Authors:** Abdullah Alatawi, SoonJye Kho, Michael P. Markey

**Affiliations:** ^1^Department of Biochemistry and Molecular Biology, Wright State University, Dayton, OH 45435, USA; ^2^Department of Computer Science and Engineering, Wright State University, Dayton, OH 45435, USA

## Abstract

The p53 tumor suppressor integrates upstream signals such as DNA damage and active oncogenes to initiate cell cycle arrest or apoptosis. This response is critical to halting inappropriate growth signals. As such, p53 activity is lost in cancer. In melanoma, however, the p53 gene is intact in a reported 94% of human cases. Rather than direct mutation, p53 is held inactive through interaction with inhibitory proteins. Here, we examine the expression of the two primary inhibitors of p53, MDM2 and MDM4, in genomic databases and biopsy specimens. We find that MDM4 is frequently overexpressed. Moreover, changes in splicing of MDM4 occur frequently and early in melanomagenesis. These changes in splicing must be considered in the design of therapeutic inhibitors of the MDM2/4 proteins for melanoma.

## 1. Introduction

As it plays a critical role in the arrest of inappropriate cell growth, the p53 tumor suppressor gene (TP53) is mutated in approximately half of all human cancers [1]. In fact, it was mutational profiling of TP53 in squamous cell skin cancer that first implicated UV-induced dipyrimidine photoproducts as oncogenic [2]. However, it has been reported that TP53 is mutated in only 6% of melanomas [3]. Lying at a critical junction between DNA damage sensing and arrest or cell death, it is essential for a cancer cell to therefore find another way to repress the activity of p53. Commonly, this is through expression of the primary inhibitors of p53: MDM2 and MDM4. Both are able to bind to p53 and prevent its ability to transactivate target genes [4–7], but only MDM2 acts as a ubiquitin ligase to target p53 for proteasomal destruction [8–10]. On the contrary, MDM4 overexpression is observed in many cancers [11], including melanomas.

Ensembl contains at least 17 different transcripts derived from the MDM4 gene, five of which have level 1 evidence [12]. Interestingly, these transcripts result from alternative splicing which includes or excludes exons at different functional regions of the MDM4 gene. For example, the transcripts known as MDM4-211, MDM4-G, and MDM4-XALT2 all lack portions of the p53-binding domain while retaining the RING domain, through which MDM4 heterodimerizes with MDM2 [13–15]. Conversely, MDM4-S and MDM4-XALT1 retain the p53-binding domain but lack the RING domain [14–16]. Clearly, the biological impact of MDM4 overexpression will depend on which transcripts are being expressed. Despite the importance of MDM4 in melanoma, however, there has never been a systematic study of which transcripts are present in human melanomas.

## 2. Materials and Methods

  Analysis of publicly available data: data were obtained from several public resources. Gene mutation frequency in melanoma was taken from the dataset SKCM-US, a 466-subject study of melanoma patients in the United States [17], and analyzed at the International Cancer Genome Consortium data portal [18]. The results published here are in whole or part based on data generated by the TCGA Research Network (https://www.cancer.gov/tcga). TCGA copy number data for SKCM were analyzed using Oncomine [19]. TCGA survival data for SKCM were analyzed using OncoLnc [20]. Patient specimen expression data (RSEM RNAseqV2 normalized reads) were separated into the highest versus lowest quartiles of MDM4 expression. Survival data for these samples were used to construct a Kaplan–Meier plot. Isoform-specific expression data for MDM4 in normal skin were analyzed in the Genotype-Tissue Expression (GTEx) data portal [21]. The Genotype-Tissue Expression (GTEx) Project was supported by the Common Fund of the Office of the Director of the National Institutes of Health and by NCI, NHGRI, NHLBI, NIDA, NIMH, and NINDS. The data used for the analyses described in this manuscript were obtained from the GTEx portal and dbGaP accession number phs000424.v8.p2 on 5/29/2020. For melanomas, isoform-specific expression data were obtained from the Patient-Derived Model Repository (PDMR) [22]. TPM isoform data were filtered for disease body location “skin” plus CTEP SDC description “melanoma.” These data have been mapped to the human transcriptome based on exon models from hg19 using Bowtie 2 (version 2.2.6 [23]). SAM files were converted to BAM using SAMtools [24], and the coordinates were converted to the genomic (hg19) coordinates using RSEM (version 1.2.31 [25]). RSEM was also used for gene and transcript quantifications. Because some transcript identifiers from hg19 map to the same RefSeq, the IsoPct (percentage of a sample's transcripts that were each specific transcript) was collapsed such that the sum of any sample's MDM4 transcript data is 100%. USCS Genome Browser isoform identifiers were matched to transcripts modeled in Ensembl in order to compare with GTEx data. Kaplan–Meier analysis of TCGA data for specific isoforms of MDM4 was performed using psichomics [26] in Bioconductor.
  Specimens: a total of 40 formalin-fixed, paraffin-embedded (FFPE) specimens (30 malignant melanomas and 10 benign melanocytic nevi) were collected from American Dermatopathology Laboratory (Springboro, Ohio, USA). Average patient age was 54.1 ± 19.5 years. Age, sex, tumor location, diagnosis, and other clinical diagnostic details are given in Supplementary Table 1.
  RNA extraction*:* RNA was extracted from FFPE specimens using the truXTRAC FFPE RNA Kit (Covaris, Woburn, MA) and a Covaris M220 Ultrasonicator following the manufacturer's protocol. Once the RNA purification process was completed, sample RNA was quantified by NanoDrop and stored immediately at −80°C.
  RT-PCR: 500 ng of RNA was reverse transcribed with 50 ng/*µ*l random hexamers using the SuperScript IV CellsDirect cDNA Synthesis Kit (Applied Biosystems). PCR was carried out using GoTaq^®^ Green Master Mix (Promega, Madison, WI, USA) with 25 *µ*l reaction volumes. PCR was performed for 2 minutes at 95°C; then, 30 cycles of 30 sec at 95°C; 30 sec at 55°C; 30 sec at 73°C; 5 minutes at 73°C. Products were separated on a 2% agarose TBE gel, with SYBR safe stain and a 50 bp ladder (Thermo Fisher Scientific, Waltham, MA, USA). Gels were run on 100 V constant for approximately 60 minutes. Gels were imaged on Amersham^™^ Imager 600 (Amersham, Little Chalfont, United Kingdom). Beta-actin was used as an amplification control. PCR primers are given in Supplementary Table 2. A sample was counted as positive for an isoform if its PCR amplicon was present, beta-actin positive control was present, and a no-DNA negative control was negative.

## 3. Results and Discussion

**Figure 1:**
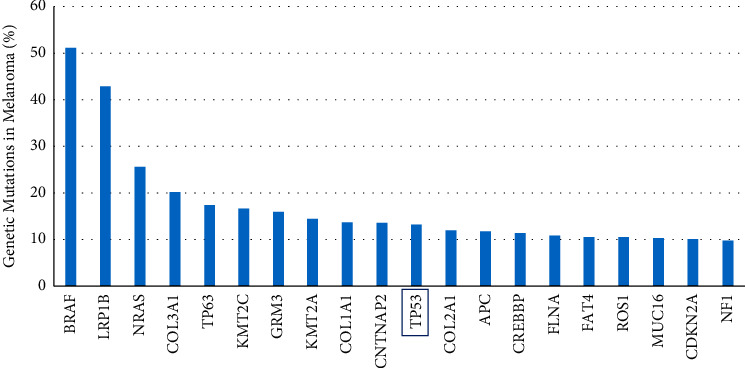
Most melanomas have an intact p53 gene. Tumors from 466 melanoma subjects in the United States were tested for simple somatic mutations as part of the TCGA study SKCM-US [17]. Genes were ranked by the frequency of mutation. Top 20 results are shown.

**Figure 2:**
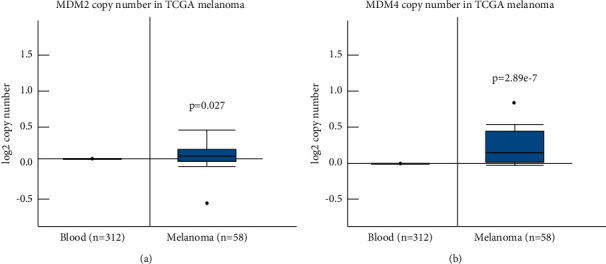
MDM4 is frequently amplified in melanoma, in contrast with MDM2. Skin cutaneous melanoma data from TCGA with copy number data available for tumor and matched normal tissue (blood) were analyzed for amplification or deletion of the genes MDM2 (a) and MDM4 (b). Copy number is relative to the control blood specimens.

Genes mutated in melanomas were ranked in TCGA by frequency. The top 20 results are shown in Figure 1. Consistent with previous reports [3], most melanomas (87%) were found to retain an intact p53 gene. This emphasizes the need to repress p53 activity in these cancers. Therefore, we examined the frequency of amplification for both MDM2 and MDM4 in copy number data of TCGA melanomas versus normal controls (blood specimens). MDM2 copy number relative to controls is very similar (fold change: 1.099, *p*=0.027). In contrast, MDM4 was amplified both in greater magnitude (fold change: 1.148) and significance (*p*=2.89 × 10^−7^) (Figure 2).

**Figure 3:**
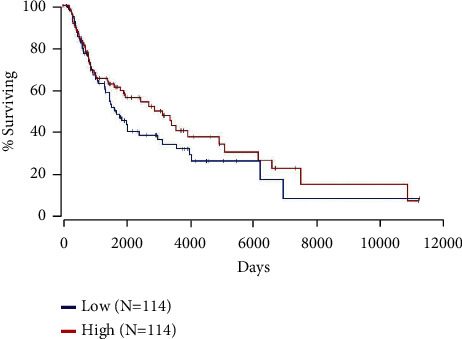
MDM4 expression does not significantly correlate with patient survival. TCGA survival data for skin cutaneous melanoma were separated by the expression of MDM4. The “High” group (red) had the expression of MDM4 in the top 25% of available subjects. “Low” (blue) subjects were in the bottom 25% of expression for MDM4. *N* for each group is 114 subjects. Kaplan–Meier analysis indicates no significant difference in survival between these groups; log-rank *p* value = 0.247.

In order to determine if MDM4 amplification is likely to result in biologically significant expression changes, the expression of MDM4 was used to separate TCGA samples with survival data into two groups: those within the highest quartile of MDM4 expression and those in the lowest quartile. As shown in Figure 3, total MDM4 expression as measured here did not correlate with survival.

**Figure 4:**
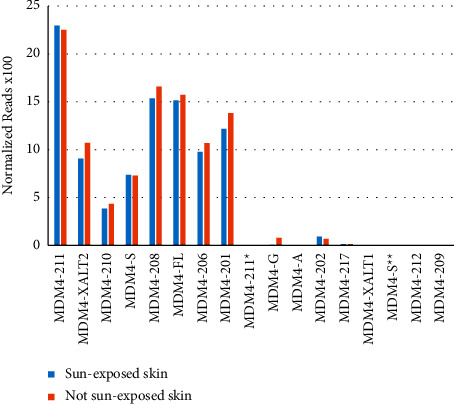
MDM4 isoform expression in normal skin. Non-sun-exposed (suprapubic) skin specimen data from 604 subjects and sun-exposed (lower leg) skin specimens from 701 subjects were analyzed in the GTEx data portal. Normalized reads × 100 for each indicated isoform of MDM4 are displayed (*y*-axis). Isoforms are labeled using their common names or Ensembl numbers. Ensembl transcript evidence level 1 (TSL1) transcripts are in bold. ^*∗*^This Ensembl transcript (ENST00000470908.5) shares a name with the transcript commonly called MDM4-211 (ENST00000367183.7). ^*∗∗*^Two transcripts encode MDM4-S.

These expression data from TCGA suggest a selective advantage for the cancer to have higher MDM4 expression but fail to demonstrate a dramatic impact on patient survival. One possible reason for this is that the small reads of next-generation sequencing used to generate these data have difficulties distinguishing between the expression of different mRNA isoforms. Unless a read falls across a unique splice junction, it is difficult to assign it to a specific isoform by NGS. Analysis of splice junctions, therefore, was carried out in the Genotype-Tissue Expression (GTEx) data portal (Figure 4). Isoform-specific expression data for MDM4 in normal skin show a dramatic variation in expression between MDM4 isoforms. MDM4-211 [13, 14] was the most commonly observed isoform, more so than the full-length isoform. Other transcripts that currently exist only in Ensembl (but not the cancer literature) were also observed, such as MDM4-208, MDM4-201, and MDM4-206. There were no significant differences observed between sun-exposed and non-sun-exposed skin.

**Figure 5:**
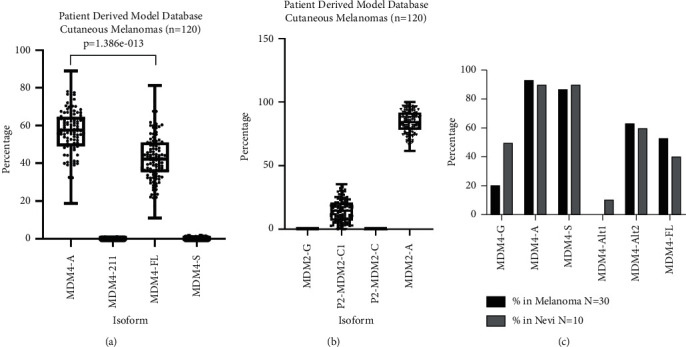
MDM4 isoform expression in melanocytic lesions is dramatically different from normal skin. Isoform-specific expression data were compared for 120 patient-derived xenografts. (a) Expression of the alternative transcript MDM4-A is significantly greater than the expression of the full-length MDM4 transcript in these specimens (Students' *t*-test *p* value = 1.386 × 10^−13^). (b) In contrast, MDM2 expression is almost entirely the full-length transcript designated as MDM2-A. (c) Analysis of 30 clinical melanoma specimens and 10 melanocytic benign nevi by isoform-specific RT-PCR.

In order to compare these isoform expression data in normal skin to melanoma, the Patient-Derived Model Repository was queried for isoform-specific MDM4 expression (Figure 5). In these primary cultures of human melanomas, only two isoforms of MDM4 were identified in most samples: full-length mRNA (MDM4-FL) and MDM4-A. MDM4-211 and MDM4-S were infrequently observed. Surprisingly, MFM4-A was significantly more common than any other isoform, including MDM4-FL (Figure 5(a)). For comparison, the same analysis was performed for the related gene MDM2. In contrast to MDM4, almost 100% of the samples expressed the full-length MDM2 isoform (Figure 5(b)). These NGS results were compared to isoform-specific RT-PCR for clinical melanoma and melanocytic nevi specimens. As in the NGS data, MDM4-A was the most frequently observed isoform. However, MDM4-S was also common. These differences are likely due to different methods used; RT-PCR for specific splice junctions is highly specific and will return a positive result with even low levels of target mRNA present. Interestingly, melanocytic nevi strongly resembled melanomas in their MDM4 isoform profiles. These data suggest that the splicing changes between normal skin (Figure 4) and melanomas (Figure 5) are already in place at the stage of nevi formation.

**Figure 6:**
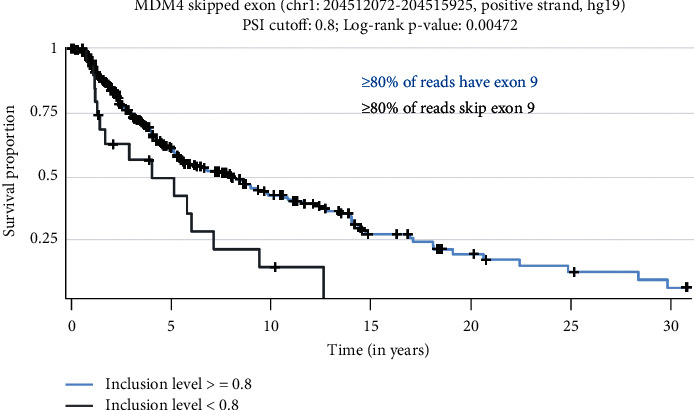
MDM4-A isoform correlates with poor survival. The subjects with an exon 9 percent spliced in (PSI) greater than 0.8 (indicating inclusion of exon 9 seen in ≥80% of transcripts, *n* = 323) had significantly higher survival than subjects where ≥80% of transcripts skipped exon 9 (*n* = 22, log-rank *p* value = 0.00472).

Studies of MDM4-S have recently suggested that the primary function of this splice variant is to decrease the levels of MDM4-FL by diverting pre-mRNA toward exon 9 skipping. The MDM4-S protein is rarely detectable without inhibition of proteasomal degradation [27]. Similarly, a protein product of MDM4-A has not been described in the literature. The transcript was first observed in the cervical cancer cell line C33a [28] and later described in gliomas [29]. Lacking the acidic domain, MDM4-A protein may be targeted by MDM2 for ubiquitination and degradation. In order to determine if MDM4-A mRNA expression is likely to have a biological impact in melanoma, TCGA samples with exon expression and survival data were analyzed based on the inclusion of MDM4 exon 9 (chr1: 204512072–204515925) using the psichomics package in R (Figure 6). Skipping of exon 9 indicates MDM4-A. Of 345 subjects in the analysis, only 6.4% had at least 80% of their MDM4 reads skipping exon 9. However, these subjects had significantly lower survival (*p*=0.00472).

## 4. Conclusions

Findings from TCGA skin cancer samples (Figure 1) are consistent with reports that p53 is mutated in only 6% of melanomas [3]. The tumor-suppressive activities of the p53 protein are therefore repressed by other means. MDM4 is known to be highly expressed in the skin [30]. We observed that MDM4 is amplified in melanomas, more so than the related protein MDM2 (Figure 2).

**Figure 7:**
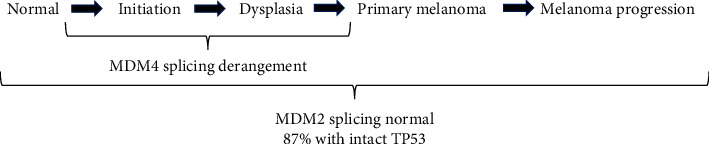
Model of melanomagenesis with the timeline of MDM2/4/p53 changes. Throughout progression from normal skin to metastatic disease, most cases retain wild-type p53 and demonstrate normal splicing and normal copy numbers of MDM2. In contrast, changes in MDM4 splicing are already in place in melanocytic nevi. MDM4 tends to be amplified in melanomas, and the profile of mRNA isoforms expressed is dramatically different from normal skin. This includes relatively high levels of MDM4-A.

However, higher total expression of MDM4 did not correlate with melanoma patient survival in these TCGA data (Figure 3). We wanted to tease apart MDM4 isoform expression from total MDM4 expression. First, this was performed for expression data in normal skin, with and without sun exposure (Figure 4). These data present a surprising variety of MDM4 splice isoforms in normal skin, regardless of sun exposure. The MDM4-211 unique splice junction was observed most frequently, followed by MDM4-208 (ENST00000462012.1) and then MDM4-FL. When compared to data from short-term cultures of human melanomas, the pattern of MDM4 isoform expression is dramatically different (Figure 5(a)). By the time of clinical excision, MDM4-A has become the most common isoform. MDM4-211 and MDM4-S are rarely detected. This is consistent with the proposed role of MDM4-S in decreasing total MDM4-FL expression, allowing p53 activity. Similarly, a proposed role for MDM4-211 is the stabilization of MDM2. MDM4-211 retains the RING finger allowing binding to MDM2 whilst lacking the p53-binding domain. This stabilization of MDM2 may promote degradation of p53 [13]. These changes in MDM4 isoform expression had no parallels in MDM2 (Figure 5(b)). MDM4-A expression could be a consequence of the tumor culturing process used to generate the PDMR data. Therefore, isoform-specific RT-PCR was used to confirm the expression of MDM4-A in patient melanoma specimens, as well as in precancerous melanocytic nevi. The transition to MDM4-A expression may therefore represent an early event in melanomagenesis (Figure 7).

As with MDM4-S, a splicing change need not result in the expression of a novel protein in order to have a biological impact [31]. The diversion of MDM4 pre-mRNA away from the full-length isoform and toward unstable isoforms such as MDM4-S could reduce total MDM4 protein available to inhibit p53, allowing MDM4-S to lead to p53 activation. However, we observe a negative impact of MDM4-A expression on survival, and this would suggest that MDM4-A does, in fact, result in the expression of an oncogenic protein. The expression of this protein remains to be demonstrated, along with a mechanism for this purported oncogenic activity.

Inhibiting MDM4 in melanoma arrests the cell cycle, and this is not rescued by the inhibition of p53 [32]. Therefore, altering the splicing of MDM4 toward unstable isoforms is an attractive target for therapy development. If the MDM4-A isoform is oncogenic in melanomas as suggested here, care must be taken to not shift alternative splicing toward this isoform.

## Data Availability

The underlying data are presented within the manuscript itself, in the supplementary tables, or freely available within the databases referenced in the manuscript. These include the International Cancer Genome Consortium, The Cancer Genome Atlas, and the Genotype-Tissue Expression (GTEx) Project as indicated in Section 2.

## Supplementary Materials

Supplementary Materials Supplementary Table 1: clinical diagnostic details of specimens used for RT-PCR analysis. Supplementary Table 2: PCR primers.
