# Supplementary material for: MDM4 Isoform Expression in Melanoma Supports an Oncogenic Role for MDM4-A
Source: J Skin Cancer. 2021 Oct 16;2021:3087579. doi: 10.1155/2021/3087579 (PMC8541850; doi:10.1155/2021/3087579)
Supplement: Supplementary Materials — Supplementary Table 1: clinical diagnostic details of specimens used for RT-PCR analysis. Supplementary Table 2: PCR primers. [file 3087579.f1.zip › 3087579.f1/Description of Supplemental Files.pdf]

Description of Supplemental Files

Supplemental Table 1. Clinical diagnostic details of specimens used for RT-PCR analysis.

Supplemental Table 2. PCR primers.
