# Supplementary material for: MDM4 Isoform Expression in Melanoma Supports an Oncogenic Role for MDM4-A
Source: J Skin Cancer. 2021 Oct 16;2021:3087579. doi: 10.1155/2021/3087579 (PMC8541850; doi:10.1155/2021/3087579)
Supplement: Supplementary Materials — Supplementary Table 1: clinical diagnostic details of specimens used for RT-PCR analysis. Supplementary Table 2: PCR primers. [file 3087579.f1.zip › 3087579.f1/Supp Table 1.pdf]

Cases	Patient Age	Patient Sex	Bioposy Site	Diagnosis	Breslow Thickness (mm)	Assoc or Nevus (Y or N)	Castle Class
Case 1	49	M	Right Upper Back PS	melanoma	0.5	Y	1A
Case 2	55	M	Right Back	melanoma	1.5	N	1B
Case 3	67	M	Left Ear	melanoma	1.6	N	1A
Case 4	50	M	Left Medial Calf	melanoma	2.5	N	2B
Case 5	53	M	Right Superior Upper Back	melanoma	0.4	N	1A
Case 6	33	F	Left Posterior Shoulder	melanoma	0.4	Y	1A
Case 7	53	M	Right Inner Tight	melanoma	0.8	N	1B
Case 8	68	M	Nose	melanoma	0.5	N	2A
Case 9	64	M	Right Calf	melanoma	0.6	N	1B
Case 10	74	M	Left Forearm Proximal	melanoma	2.1	N	2B
Case 11	70	M	Left Upper Arm	melanoma	0.3	N	1A
Case 12	71	M	Right Posterior Sholuder	melanoma	0.3	N	1A
Case 13	71	M	Left Forearm Proximal	melanoma	1.5	N	2B
Case 14	29	F	Right Upper Helix	melanoma	0.5	N	1A
Case 15	39	M	Right Anterior Deltoid	melanoma	1.1	Y	1A
Case 16	58	F	Left upper arm	melanoma	0.9	N	1A
Case 17	63	F	Left Upper Arm	melanoma	0.9	Y	1A
Case 18	83	M	Scalp	melanoma	1.7	N	2A
Case 19	73	M	Vertex	melanoma	0.5	N	1A
Case 20	67	M	Right Arm	melanoma	0.4	Y	1A
Case 21	43	F	Right Abdomen	melanoma	0.4	Y	1A
Case 22	61	F	Right thigh	melanoma	0.9	Y	1A
Case 23	68	F	Left anterior proximal thigh	melanoma	0.7	N	1A
Case 24	73	F	Right upper posterior arm	melanoma	2.5	N	2B
Case 25	71	F	Left posterier shoulder	melanoma	0.4	N	1A
Case 26	79	M	Right Posterior Sholuder	melanoma	1.2	N	2A
Case 27	57	F	Left posterior deltoid	melanoma	0.5	N	1A
Case 28	47	F	Top scalp	melanoma	4.1	N	2B
Case 29	70	M	Right lower leg	melanoma	0.8	N	1A
Case 30	77	M	Back of neck	melanoma	5	N	2B
Case 31	14	F	Right T4	Compound melanocytic nevus			
Case 32	32	F	Left temple	Compound melanocytic nevus			
Case 33	34	F	Right antecubital	Compound melanocytic nevus			
Case 34	60	F	Left mid. Lat. Low. Extremity	Spindle cell nevus of reed, inflamed and irritated			
Case 35	2	M	Might medial forearm	Pigmented spindle and epitehlioid cell melanocytic nevus, inflamed			
Case 36	49	F	Left medial thigh	Intradermal melanocytic nevus			
Case 37	41	F	Right mid. Inframammary fold	Intradermal melanocytic nevus			
Case 38	54	F	right alar crease	Intradermal melanocytic nevus			
Case 39	15	F	right dorsal forearm	Spitz's Nevus, inflamed			
Case 40	25	F	Right posterior thigh	Spitz's Nevus			
