# Supplementary material for: MDM4 Isoform Expression in Melanoma Supports an Oncogenic Role for MDM4-A
Source: J Skin Cancer. 2021 Oct 16;2021:3087579. doi: 10.1155/2021/3087579 (PMC8541850; doi:10.1155/2021/3087579)
Supplement: Supplementary Materials — Supplementary Table 1: clinical diagnostic details of specimens used for RT-PCR analysis. Supplementary Table 2: PCR primers. [file 3087579.f1.zip › 3087579.f1/Supp Table 2.pdf]

Amplicon	Forward Primer (5'-3')	Reverse Primer (5'-3')
MDM4-FL	*AGATGCTGCTCAGACTCTCG*	*TCAGGATGTGGGTACTGCCA*
MDM4-211	*CTCCTGGACAAATCAATCAGGAAA*	*TGATCCCTGCAACTCAGTGG*
MDM4-XAlt1	*CAGGTGCGCAAGGTGAAATG*	*ACTACAGGTGATTGAAGTGGGA*
MDM4-XAlt2	*ACTGTTAAAGAGGTGATTGAAGTGG*	*TGATCCCTGCAACTCAGTGG*
MDM4-A	*CACACTGCCTACCTCAGAGC*	*GACAAATCAGGTGATTGAAGTGGG*
MDM4-G	*CCTGGACAAATCAATCAGGATCAC*	*TATCCCCACACTGCCTACCT*
MDM4-S	*CAGCAGGTGCGCAAGGTGAA*	*CACTGCTACTACAGCAAAGTG*
B-Actin	*TTCCTATGTGGGCGACGAG*	*CGTGTGGCTCCCGAGGA*
